# Supplementary material for: Deletion of wfs1 Impairs Oligodendrocyte Precursor Cells Dorsal Distribution and Myelination Through the wfs1-hmgcs1 Axis in Zebrafish
Source: Biology (Basel). 2026 Aug 16;15(16):1402. doi: 10.3390/biology15161402 (PMC13510151; doi:10.3390/biology15161402)
Supplement: Supplementary file 1 [file biology-15-01402-s001.zip › File S1-Transcriptome sequencing data and differentially expressed genes (DEGs) in wfs1-- zebrafish at 5 dpf/1.GO/wfs1KOvsWT/GSEA/wfs1KO/PROTEIN_TARGETING_TO_ER/PROTEIN_TARGETING_TO_ER(GO_0045047).html]

Details for gene set PROTEIN\_TARGETING\_TO\_ER(GO:0045047)[GSEA]


|  || Dataset | deg.sample |
| Phenotype | sample.cls |
| Upregulated in class | wfs1KO |
| GeneSet | PROTEIN\_TARGETING\_TO\_ER(GO:0045047) |
| Enrichment Score (ES) | 0.49789286 |
| Normalized Enrichment Score (NES) | 2.1468186 |
| Nominal p-value | 0.0 |
| FDR q-value | 0.023679694 |
| FWER p-Value | 0.097 |
Table: GSEA Results Summary

  

Fig 1: Enrichment plot: PROTEIN\_TARGETING\_TO\_ER(GO:0045047)
  
Profile of the Running ES Score & Positions of GeneSet Members on the Rank Ordered List

  

| PROBE | DESCRIPTION   (from dataset) | GENE SYMBOL | GENE\_TITLE | RANK IN GENE LIST | RANK METRIC SCORE | RUNNING ES | CORE ENRICHMENT || 1 | 266757 | tram1 |  |  | 390 | 3.317 | 0.2504 | Yes |
| 2 | 337190 | ssr3 |  |  | 441 | 3.145 | 0.4979 | Yes |
| 3 | 325704 | asna1 |  |  | 4026 | 0.824 | 0.4506 | No |
| 4 | 436845 | srprb |  |  | 5930 | 0.594 | 0.4380 | No |
| 5 | 436861 | sec63 |  |  | 6659 | 0.523 | 0.4566 | No |
| 6 | 266796 | sec61a1l |  |  | 9478 | 0.335 | 0.3947 | No |
| 7 | 446119 | srp68 |  |  | 10323 | 0.283 | 0.3906 | No |
| 8 | 541528 | zgc:113278 |  |  | 11744 | 0.204 | 0.3622 | No |
| 9 | 192298 | sec61a1 |  |  | 13031 | 0.138 | 0.3328 | No |
| 10 | 393942 | efhc1 |  |  | 13153 | 0.132 | 0.3395 | No |
| 11 | 393963 | srp54 |  |  | 13988 | 0.093 | 0.3207 | No |
| 12 | 336867 | sgta |  |  | 14590 | 0.066 | 0.3070 | No |
| 13 | 431772 | sgtb |  |  | 15568 | 0.019 | 0.2779 | No |
| 14 | 405884 | spcs3 |  |  | 18297 | -0.089 | 0.1993 | No |
| 15 | 563660 | sec62 |  |  | 18670 | -0.107 | 0.1961 | No |
| 16 | 393489 | zfand2a |  |  | 19661 | -0.151 | 0.1770 | No |
| 17 | 541342 | spcs2 |  |  | 20221 | -0.176 | 0.1734 | No |
| 18 | 394071 | srp9 |  |  | 21127 | -0.222 | 0.1626 | No |
| 19 | 327335 | srp72 |  |  | 21356 | -0.234 | 0.1739 | No |
| 20 | 569063 | srpr |  |  | 21485 | -0.240 | 0.1889 | No |
| 21 | 791986 | sec61b |  |  | 21981 | -0.266 | 0.1945 | No |
| 22 | 406344 | tram2 |  |  | 22900 | -0.319 | 0.1910 | No |
| 23 | 406652 | srp19 |  |  | 22993 | -0.327 | 0.2140 | No |
| 24 | 606666 | spcs1 |  |  | 23971 | -0.390 | 0.2142 | No |
| 25 | 619260 | srp14 |  |  | 24740 | -0.433 | 0.2244 | No |
Table: GSEA details
[plain text format]

  

Fig 2: PROTEIN\_TARGETING\_TO\_ER(GO:0045047)
  
Blue-Pink O' Gram in the Space of the Analyzed GeneSet

  

Fig 3: PROTEIN\_TARGETING\_TO\_ER(GO:0045047): Random ES distribution
  
Gene set null distribution of ES for
 **PROTEIN\_TARGETING\_TO\_ER(GO:0045047)**
